# Contributions of blood–brain barrier imaging to neurovascular unit pathophysiology of Alzheimer’s disease and related dementias

**DOI:** 10.3389/fnagi.2023.1111448

**Published:** 2023-02-09

**Authors:** Yuto Uchida, Hirohito Kan, Keita Sakurai, Kenichi Oishi, Noriyuki Matsukawa

**Affiliations:** ^1^The Russell H. Morgan Department of Radiology and Radiological Science, Johns Hopkins University School of Medicine, Baltimore, MD, United States; ^2^Department of Integrated Health Sciences, Nagoya University Graduate School of Medicine, Nagoya, Japan; ^3^Department of Radiology, National Center for Geriatrics and Gerontology, Ōbu, Aichi, Japan; ^4^Department of Neurology, Nagoya City University Graduate School of Medical Sciences, Nagoya, Japan

**Keywords:** Alzheimer’s disease, biomarker, blood–brain barrier, MRI, neurovascular unit

## Abstract

The blood–brain barrier (BBB) plays important roles in the maintenance of brain homeostasis. Its main role includes three kinds of functions: (1) to protect the central nervous system from blood-borne toxins and pathogens; (2) to regulate the exchange of substances between the brain parenchyma and capillaries; and (3) to clear metabolic waste and other neurotoxic compounds from the central nervous system into meningeal lymphatics and systemic circulation. Physiologically, the BBB belongs to the glymphatic system and the intramural periarterial drainage pathway, both of which are involved in clearing interstitial solutes such as β-amyloid proteins. Thus, the BBB is believed to contribute to preventing the onset and progression for Alzheimer’s disease. Measurements of BBB function are essential toward a better understanding of Alzheimer’s pathophysiology to establish novel imaging biomarkers and open new avenues of interventions for Alzheimer’s disease and related dementias. The visualization techniques for capillary, cerebrospinal, and interstitial fluid dynamics around the neurovascular unit in living human brains have been enthusiastically developed. The purpose of this review is to summarize recent BBB imaging developments using advanced magnetic resonance imaging technologies in relation to Alzheimer’s disease and related dementias. First, we give an overview of the relationship between Alzheimer’s pathophysiology and BBB dysfunction. Second, we provide a brief description about the principles of non-contrast agent-based and contrast agent-based BBB imaging methodologies. Third, we summarize previous studies that have reported the findings of each BBB imaging method in individuals with the Alzheimer’s disease continuum. Fourth, we introduce a wide range of Alzheimer’s pathophysiology in relation to BBB imaging technologies to advance our understanding of the fluid dynamics around the BBB in both clinical and preclinical settings. Finally, we discuss the challenges of BBB imaging techniques and suggest future directions toward clinically useful imaging biomarkers for Alzheimer’s disease and related dementias.

## 1. Introduction

The blood–brain barrier (BBB) maintains brain homeostasis through sophisticated anatomical and physiological systems. The BBB is composed of endothelial cells of the capillary wall, pericytes embedded in the endothelial basement membrane, and astrocytic end-feet that surround the parenchymal basement membrane, known overall as the glia limitans (Engelhardt and Sorokin, 2009). Tight junction proteins seal the endothelial cells together and restrict the intrusion of bloodstream substances into the central nervous system (Ballabh et al., 2004). The BBB is also a highly selective semipermeable border, which allows the passive diffusion of some hydrophobic molecules such as oxygen, carbon dioxide, and hormones, as well as facilitates the selective and active transport of water, ions, organic anions, and hydrophilic macromolecules such as glucose and amino acids that are vital nutrients to neurons (Obermeier et al., 2013). Any neuron is less than 10–20 μm away from a capillary (Tsai et al., 2009), which implies that every neuron has its own capillary. This demonstrates the critical relationship between the vascular and neuronal compartments, called the neurovascular unit (NVU; Montagne et al., 2016; Chagnot et al., 2021).

For imaging analysis of the BBB, the most commonly used approaches are to track the uptake of intravenously injected tracers as they leak from the bloodstream into the brain through the BBB. In rodents, two-photon microscopy can monitor the leakage of fluorescent dyes across the BBB, but the field of view is small, and scanning depth is limited (Burgess et al., 2014; Dickie et al., 2020). Photoacoustic imaging enables visualization of dyes or probes with specific absorption characteristics at greater depths than fluorescence-based imaging systems, but is still unable to provide adequate penetration for human brains and is difficult to quantify (Beard, 2011). For human subjects, positron emission tomography (PET) can quantitatively measure the activity of BBB-specific transporters (Piert et al., 1996; Syvänen and Eriksson, 2013), but has lower spatial resolution among the conventional imaging modalities and requires blood sampling during the scan. In addition, repeat scanning in at-risk healthy populations for longitudinal aging and individuals with cognitive decline is difficult to justify due to the cumulative dose of ionizing radiation even if it has little effect on the participants’ health (Nasrallah et al., 2013).

Hence, non-invasive methods with which to detect BBB function for *in vivo* human subjects are needed to reveal the impact of BBB dysfunction on the pathogenesis and progression of Alzheimer’s disease (AD) pathological conditions. We emphasize that *in vivo* human experiments are essential to assess the BBB physiology because the fluid dynamics around the BBB would cease in *ex vivo* human brain tissues. In terms of its non-invasiveness and convenience in a clinical setting, magnetic resonance imaging (MRI) fits well with clinical research to investigate to what extent the BBB functions would be altered along with aging and Alzheimer’s pathological processes. Therefore, we focused on the magnetic resonance-based BBB imaging methodologies in this review. First, we give an overview of the relationship between Alzheimer’s pathophysiology and BBB dysfunction. Second, we provide a brief description about the principles of non-contrast agent-based and contrast agent-based BBB imaging methodologies. Third, we summarize previous studies that have reported the findings of each BBB imaging method in individuals with the AD continuum. Fourth, we introduce a wide range of AD pathophysiology in relation to BBB imaging technologies to advance our understanding of the fluid dynamics around the BBB in both clinical and preclinical settings. Finally, we discuss the challenges of BBB imaging techniques and suggest future directions toward clinically useful imaging biomarkers for AD and related dementias.

## 2. Alzheimr’s pathopyhsiology in relation to blood–brain barrier dysfunction

There is increasing evidence that supports the involvement of BBB dysfunction in the early stages of Alzheimer’s disease (AD; Montagne et al., 2015; van de Haar et al., 2016a,b; Montagne et al., 2017, 2020; Nation et al., 2019; Sweeney et al., 2019) and related dementias, such as cerebral small vessel disease (CSVD; Zhang et al., 2017; Shao et al., 2019; Wardlaw et al., 2019; Uchida et al., 2020). The pathological hallmarks of AD include the deposition of extracellular β-amyloid (Aβ) aggregates in the brain parenchyma as senile plaques and within the cerebral vessel walls and leptomeninges as cerebral amyloid angiopathy, along with intracellular hyperphosphorylated tau aggregates as neurofibrillary tangles, and neuronal cell loss as neurodegeneration (Jack et al., 2018). Recent clinicopathological and radiological data suggest that there are close relationships between BBB dysfunction and these established Alzheimer’s biomarkers (Cockerill et al., 2018; Michalicova et al., 2020; Wang et al., 2021; Ishida et al., 2022). Excessive accumulation of toxic forms of Aβ and tau proteins is assumed to result from an imbalance between its production and clearance (Tarasoff-Conway et al., 2015). Physiologically, the BBB belongs to the glymphatic system (Iliff et al., 2012, 2013; Nedergaard and Goldman, 2020) and the intramural periarterial drainage pathway (Carare et al., 2008; Weller et al., 2010; Hawkes et al., 2011; Morris et al., 2014), both of which are involved in clearing interstitial solutes such as Aβ (Cockerill et al., 2018; Wang et al., 2021) and tau proteins (Michalicova et al., 2020; Ishida et al., 2022). Further, the *ε4* allele of *APOE* gene is the strongest and most validated genetic risk factor for sporadic AD (Yamazaki et al., 2019). Emerging evidence suggests that *APOE ɛ4* directly impairs the BBB: astrocyte-secreted ApoE4 induces the degeneration of brain capillary pericytes that maintain BBB integrity (Bell et al., 2012), and individuals carrying *APOE ɛ4* are closely linked to the onset and progression of AD pathogenesis, independent of pathological Alzheimer’s biomarkers (Montagne et al., 2020; Uchida et al., 2022a).

## 3. Relationship between neurovascular unit and blood–brain barrier imaging

### 3.1. Normal neurovascular unit physiology in relation to blood–brain barrier imaging

**Figure 1:**
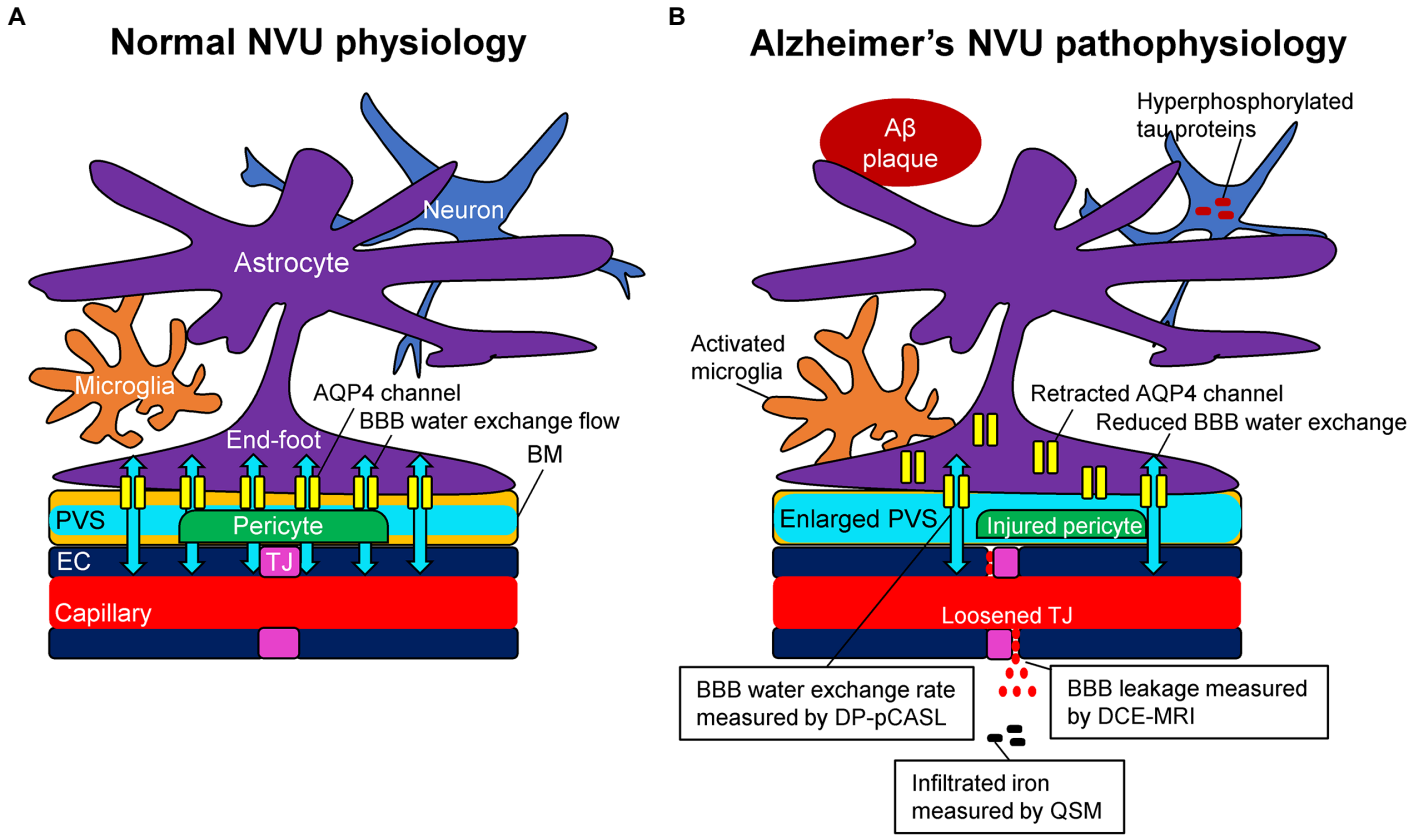
Schematic representation of the neurovascular unit (NVU) in normal physiology **(A)** and Alzheimer’s pathophysiology **(B)**. **(A)** The capillary lumen is formed by endothelial cells (EC), pericytes embedded in the basement membrane (BM), and astrocytic end-feet. Tight junction (TJ) proteins seal the ECs together and restrict the passage of solutes into the brain. The BM are separated into two layers, which are attached to the ECs and astrocytic feet, forming the perivascular space (PVS). Aquaporin-4 (AQP4) water channels expressed in the astrocytic end-feet membranes facilitate bidirectional water flow between the capillary, PVS, and interstitial tissues, resulting in the glymphatic flow. **(B)** In Alzheimer’s pathophysiology, the damaged TJ proteins loosen the seal of the ECs and provide a route for passive diffusion of toxic substances into the extravascular space, which can further damage or downregulate the NVU system. As a consequence of the injured pericyte that normally anchors the AQP4 water channels to astrocytic end-feet, the retraction of AQP4 from astrocytic end-feet membranes occurs with a reduction of bidirectional water flow, resulting in an enlarged PVS. Based on these pathophysiological changes, dynamic contrast-enhanced magnetic resonance imaging (DCE-MRI) can measure the blood–brain barrier (BBB) leakage, pseudo-continuous diffusion-prepared arterial spin labeling (DP-pCASL) can estimate the BBB water exchange rate, and quantitative susceptibility mapping (QSM) can detect the brain iron concentration, respectively.

Due to the existence of capillary endothelial cells with tight junctions, trans-endothelial permeability to plasma proteins and inorganic solutes is limited (Nitta et al., 2003; Wardlaw et al., 2003). In addition, the regulation of brain water transport is essential to brain homeostasis and its dysfunction is associated with several neurological conditions. Trans-endothelium water exchanges are through both passive (i.e., diffusion) and active (i.e., co-transport by ion pumps, carrier proteins, and transcytosis) pathways between the capillary and interstitial fluids (Oresković and Klarica, 2010). The key BBB-related water exchange pathway operates through a set of perivascular trans-membrane proteins, which are called aquaporin-4 (AQP4) channels. AQP4 channels were well-regulated and localized to perivascular astrocytic end-feet, known as AQP4 polarization, and form a central pathway for the glymphatic system, facilitating water transport across the basement membrane (Ibata et al., 2011; Papadopoulos and Verkman, 2013; Ohene et al., 2019). The BBB water exchange flow through AQP4 channels plays a role in the drainage of brain metabolites and other neurotoxic compounds from the central nervous system into meningeal lymphatics and systemic circulation as a part of the glymphatic system (Iliff et al., 2012; Figure 1A).

### 3.2. Alzheimer’s neurovascular unit pathophysiology in relation to blood–brain barrier imaging

**Figure 2:**
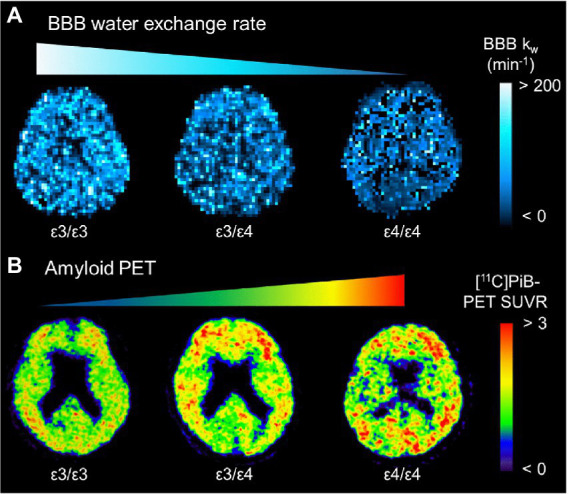
Representative BBB *k_w_* map **(A)** and [^11^C]PiB-PET SUVR **(B)** from an *APOE ɛ4* noncarrier (*ε3/ε3*), a heterozygote (*ε3/ε4*), and a homozygote (*ε4/ε4*). The *k_w_* map from the homozygote (*ε4/ε4*) displays the lowest *k_w_* values, which are associated with the highest SUVRs of [^11^C]PiB-PET. *APOE*: apolipoprotein E; BBB, blood–brain barrier; PiB, Pittsburgh compound B; SUVR, standard uptake value ratio [adapted with permission from Uchida et al. (2022a)].

**Figure 3:**
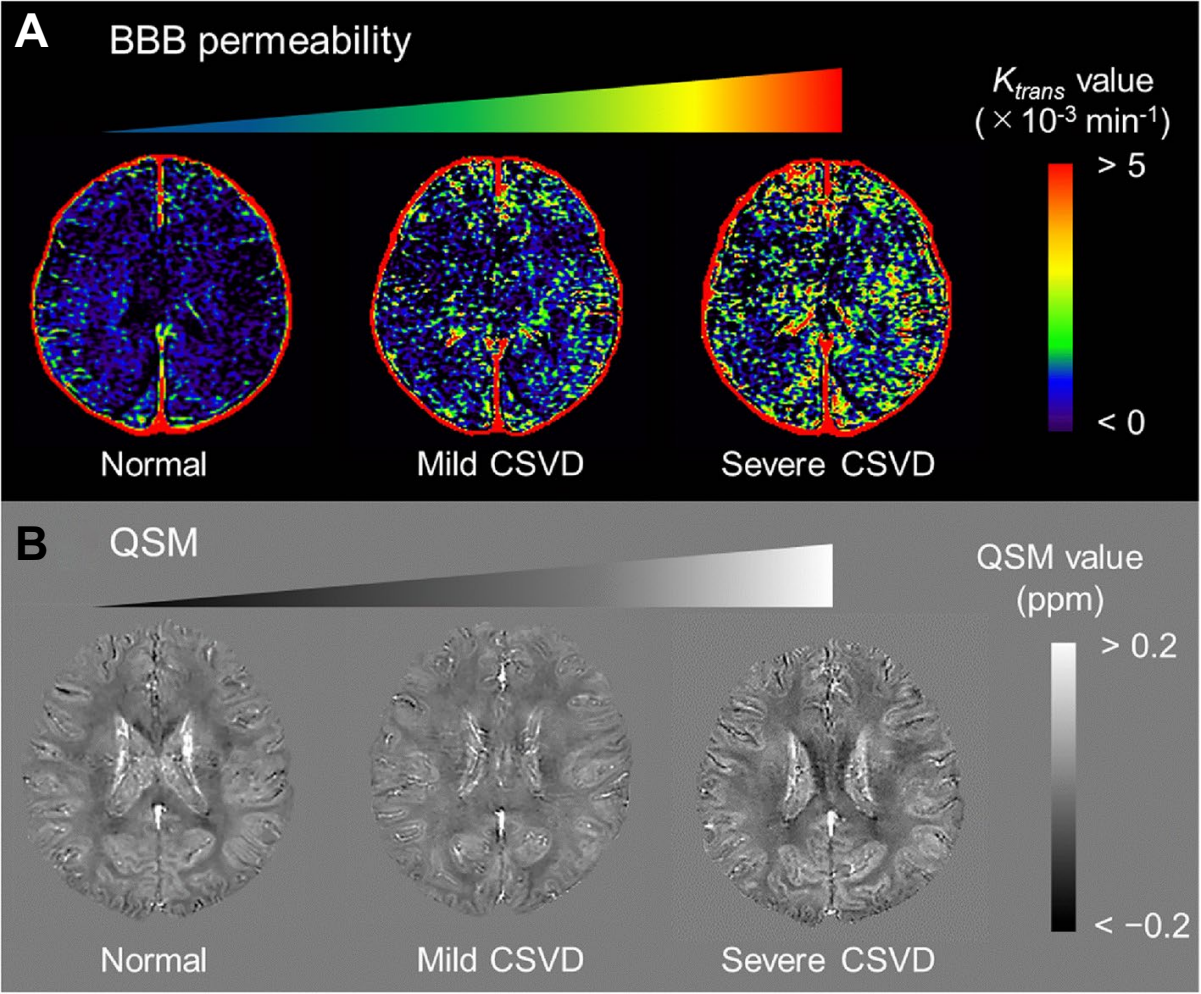
Representative BBB permeability *K_trans_* map **(A)** and QSM **(B)** from normal, mild CSVD, and severe CSVD. The *K_trans_* map from the severe CSVD displays the highest *K_trans_* values, which are associated with the highest susceptibility values. BBB, blood–brain barrier; CSVD, cerebral small vascular disease; QSM, quantitative susceptibility mapping [adapted with permission from Uchida et al., 2020].

Alzheimer’s pathological changes include NVU pathophysiology, which can be detected by BBB imaging (Figure 1B). There are mainly two key mathematical MRI models for BBB imaging, which will be discussed in this review: those that utilize contrast agents to enhance relaxation rate differences between the intravascular and extravascular compartments (Joseph and Novel, 2020); and those that utilize the dynamic properties of arterial spin labeling (ASL) to first isolate signals from intravascular spins and then estimate the water exchange rate on the evolving signals around the BBB (Dickie et al., 2020). The former model is called dynamic contrast-enhanced MRI (DCE-MRI), which requires the injection of gadolinium-based contrast agents into the vein and has been widely used to measure BBB permeability (Heye and Culling, 2014). Subtle BBB leakage, triggered by loosened endothelial tight junctions (Laurent et al., 2006; Wang et al., 2011) and injured pericytes (Montagne et al., 2015), can be detected using DCE-MRI. The latter model is called ASL-based BBB imaging, which utilizes water as an endogenous tracer alternative to contrast agents. Among ASL-based BBB imaging, a diffusion-prepared pseudo-continuous arterial spin labeling (DP-pCASL) technique has been developed to measure the water exchange rate across the BBB (St Lawrence et al., 2012; Lin et al., 2019; Shao et al., 2019). Aberrant AQP4 expression is linked with decreased efficiency of the BBB water exchange rate (Ohene et al., 2019) and results in excessive Aβ brain deposition (Figure 2; Uchida et al., 2022a). In addition to these direct mathematical BBB models, measurement of several toxic substances from blood can be regarded as an indirect biomarker for BBB dysfunction. Blood-derived substances include fibrinogen, thrombin, hemoglobin, iron-containing hemosiderin, free iron, plasmin, environmental toxins and metals, and possibly, microbial pathogens, which can have toxic neuronal effects and lead to oxidative stress and activation of the proinflammatory microglial response, resulting in the pathological changes seen in AD (Zlokovic, 2011). Quantitative susceptibility mapping (QSM) has been available as an auxiliary biomarker that reflects disease severity in AD to measure brain tissue iron concentration, which is partly due to the BBB leakage caused by the damaged NVU (Cogswell et al., 2021; Uchida et al., 2022b; Figure 3).

## 4. Modeling for blood–brain barrier imaging

### 4.1. Modeling for blood–brain barrier water exchange rate (*k_w_*)

The BBB water exchange rate, *k_w_*, can be calculated based on the capillary permeability surface-area product of water (*PS_w_*) per unit-mass tissue according to the Renkin-Crone equation (Renkin, 1959; Crone, 1963):


$$PS_{w}=-\ln\left(1-E_{w}\right)\times CBF$$

where *E_w_* is the water extraction ratio between the capillary and brain tissue compartments and *CBF* is the cerebral blood flow. To estimate *E_w_*, a long post-labeling delay (PLD) is required for complete extraction of labeled water in the brain tissue space (Lin et al., 2018; Shao et al., 2018; Uchida et al., 2022a). A single-pass approximation (SPA) model for ASL signals has been proposed to estimate the exchange rate of labeled water from the capillary into the brain tissue space, which does not account for labeled water signal contributions from the brain tissue into the capillary space during the image acquisition (St Lawrence et al., 2012). The BBB water exchange rate, *k_w_*, is defined as *PS_w_* divided by the distribution volume of water tracer in the capillary space. In a pCASL sequence with prepared diffusion sensitizing gradients of low strength, known as DP-pCASL, signals from the intravascular spins can be nulled, leaving only signals from the extravascular spins. By applying a bi-exponential diffusion signal model to utilize the difference in signal decay between the capillary and brain tissue compartments, the proportion of signals in each compartment can be determined as a function of PLD (St Lawrence et al., 2012; Shao et al., 2019):


$$\frac{\Delta M\left(t,b\right)}{\Delta M\left(t,0\right)}=A_{1}\left(t\right)e^{-bD_{1}}+A_{2}\left(t\right)e^{-bD_{2}}$$

where *ΔM(t)* is the ASL signal at any b values, *t* is the PLD, *A1* and *A2* are intravascular and extravascular labeled water fractions, *A1(t) + A2(t)* = 1, and *D_1_* and *D_2_* are the corresponding apparent diffusion coefficients, respectively. Then, we can estimate the capillary fraction, *A_1_(t)*, at only two *b* values, which are zero and a large *b* value (*b_DW_*) sufficient to suppress the vascular signal, but with minimum effect on the tissue signal:


$$A_{1}\left(t\right)\approx 1-\frac{\Delta M\left(t,b_{DW}\right)}{\Delta M\left(t,0\right)}$$

**Figure 4:**
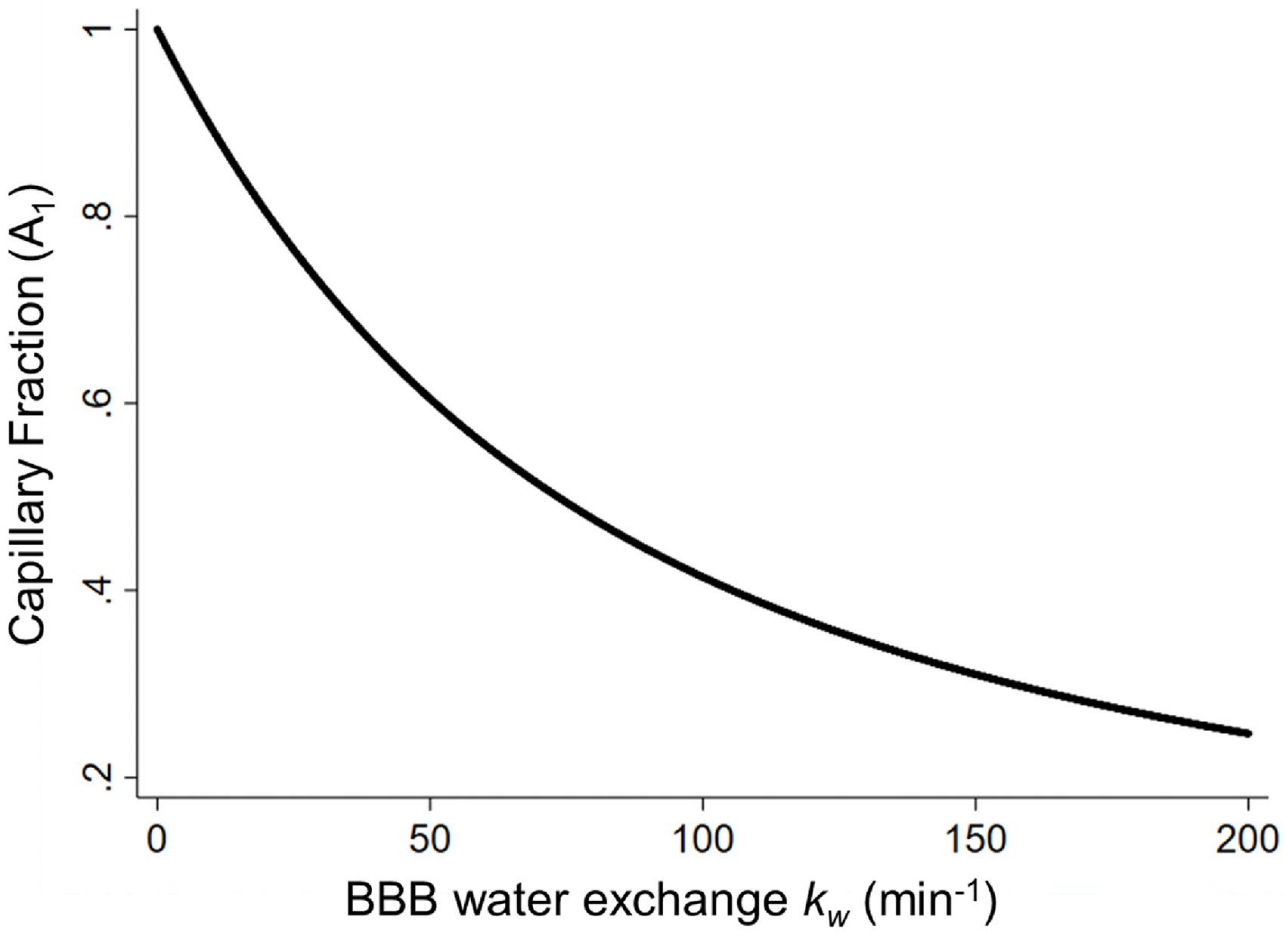
The capillary fraction of labeled water (*A*_1_) plotted as a function of the BBB water-exchange rate *k_w_* (min^−1^). Simulated data was generated using the arterial transit time, the T1 of arterial blood (1.66 s) and gray matter (1.82 s), the labeling efficiency (85%), the brain–blood partition coefficient (0.9 ml/g), and the b values (0 and 50 s/mm^2^) at a long post labeling delay (1.80 s) as inputs for the look-up table algorithm. BBB, blood–brain barrier [adapted with permission from Uchida et al. (2022a)].

The BBB water exchange rate, *k_w_*, can be extracted based on the look-up table approach between the capillary fraction, *A_1_(t)*, and *k_w_* values (Figure 4), incorporating the arterial transit time (ATT), the T1 of arterial blood and gray matter, the labeling efficiency, and the brain–blood partition coefficient as additional inputs for the SPA model (Uchida et al., 2022a). The representative *k_w_* maps are shown in Figure 2A.

### 4.2. Modeling for blood–brain barrier permeability (*K_trans_*)

The BBB permeability, *K_trans_*, is the rate at which contrast agent is delivered to the extravascular space per volume of tissue and contrast agent concentration in the blood plasma. Various pharmacokinetic models have been applied to analyze DCE-MRI data, ranging from relatively simple visual assessment of gadolinium enhancement curves to more complex fitting to pharmacokinetic models, mainly based on the Tofts and Patlak models (Heye and Culling, 2014). The conventional Tofts model assumes a bidirectional flux of tracer between the intra-and extravascular compartments with the volume transfer constant, *K_trans_*, and negligible blood volume (Tofts and Kermode, 1991). This model was extended by introducing the non-negligible blood plasma compartment as a well-mixed and highly perfused compartment (Tofts et al., 1999). The conventional Tofts model and the extended Tofts model are used for the aggressive alterations of the *K_trans_* values, such as are found in brain tumors. Meanwhile, the Patlak model assumes a unidirectional flux from the intravascular compartment into the extravascular compartment to estimate subtle BBB leakage of contrast agent to the extravascular space (Patlak et al., 1983). This two-compartment unidirectional transport model ignores the flux from the extravascular space to the intravascular space and provides *K_trans_* values as the most sensitive modeling with which to detect subtle BBB permeability, which could be applied to the Alzheimer’s NVU pathophysiology (Barnes et al., 2016). In the Patlak model, target parameters are the fractional plasma volume (*V_p_*) and the BBB permeability *K_trans_* values:


$$C_{tissue}\left(t\right)=K_{trans}\overset{t}{\underset{0}{\int }}C_{p}\left(\tau \right)d\tau +C_{p}\left(t\right)*V_{p}$$

where *C_tissue_(t)* is the contrast agent concentration of the tissue and *C_p_(t)* is determined in the arterial input function (AIF). To obtain the dynamic tracer concentration of plasma as AIF, the dynamic tracer concentration in *C_b_(t)* is converted into dynamic plasma concentration, C*_p_*(t):


$$C_{p}\left(t\right)=C_{b}\left(t\right)/\left(1-Hct\right)$$

where *Hct* is the hematocrit in the arterial blood plasma. The representative *K_trans_* maps are shown in Figure 3A.

### 4.3. Comparison between arterial spin labeling-based blood–brain barrier imaging and dynamic contrast-enhanced MRI

A number of clinicoradiological studies using the BBB water exchange rate measured by ASL-based BBB imaging, BBB permeability measured by DCE-MRI, or both have been conducted in subjects with the AD continuum. A direct comparative analysis of these methodologies found only few correlations between the BBB water exchange rate *k_w_* and BBB permeability *K_trans_* (Shao et al., 2020), suggesting that the mechanisms that regulate water exchange rate across the BBB and the BBB permeability of contrast agents are different (Table 1). In DCE-MRI, the *K_trans_* values can measure the paracellular leakage of contrast agent through the damaged endothelium and tight junctions. Meanwhile, the *k_w_* values can represent the trans-endothelium water exchange rate as both passive diffusion and active transport through ion pumps and AQP4 channels. DCE-MRI becomes more increasingly permeable to large molecules with aging (Montagne et al., 2015), particularly in patients with AD and CSVD (Farrall and Wardlaw, 2009; Zhang et al., 2017; Montagne et al., 2019). In contrast, the water exchange rate across the BBB shows a reverse trend and declines with aging (Li et al., 2005; Anderson et al., 2020). The following sections provide an overview of the relationships between each BBB imaging method and established Alzheimer’s biomarkers, as well as cognitive performance from previous reports.

**Table 1:** Comparison between ASL-based BBB imaging and DCE-MRI.

	ASL-based BBB imaging	DCE-MRI
Main MRI sequence	pCASL	Dynamic GRE
Spatial resolution (maximum)	Lower (1.9 mm × 1.9 mm × 4 mm)	Higher (0.55 mm × 0.55 mm × 5 mm)
Total scan time (minimum)	Shorter (5.53 min)	Longer (16 min)
Tracer (size)	Endogenous labelled proton (≈ 18 Da)	Exogenous gadolinium (550 Da)
Measured object	Water exchange rate across BBB	Gadolinium permeability across BBB
Mathematical model (recommended model)	Water exchange model (regularized SPA)	Pharmacokinetic model (Patlak model)
Output parameter	*k_w_*	*K_trans_*
Normal reference value among studies	Constant	Random
Intra-patient reproducibility	Good (ICC ≈ 0.75)	Good (ICC ≈ 0.75)
Pathological status (Alzheimer’s disease)	Lower *k_w_* values	Higher *K_trans_* values

ASL, arterial spin labeling; BBB, blood–brain barrier; DCE-MRI, dynamic contrast-enhanced magnetic resonance imaging; GRE, gradient echo; ICC, intra-class correlation coefficient; pCASL, pseudo-continuous arterial spin labeling; SPA, single-pass approximation.

## 5. Findings of blood–brain barrier imaging in Alzheimer’s disease subjects

### 5.1. Overview of dynamic contrast-enhanced MRI in subjects with the Alzheimer’s disease continuum

Dynamic contrast-enhanced MRI has been proven valuable in the assessment of many brain pathologies that cause BBB breakdown, such as tumors (Harrer et al., 2004; Provenzale et al., 2006; Singh et al., 2007; Mills et al., 2009; Bagher-Ebadian et al., 2012; Li et al., 2012; Zhang et al., 2012; Larsson et al., 2013), multiple sclerosis (Gaitán et al., 2011; Jelescu et al., 2011; Shinohara et al., 2011; Ingrisch et al., 2012; Cramer et al., 2014), and ischemic strokes (Kassner et al., 2009; Vidarsson et al., 2009; Thornhill et al., 2010; Topakian et al., 2010; Taheri et al., 2011). While these diseases show significant BBB breakdown, there has been growing interest in the application of DCE-MRI to pathologies associated with more subtle and chronic BBB disruption, such as CSVD (Wardlaw et al., 2008, 2009; Uchida et al., 2020), diabetes (Starr et al., 2003), and AD (Starr et al., 2009; Montagne et al., 2017). As a matter of fact, widely varying estimates of the BBB permeability *K_trans_* values were reported in each study, for each image acquisition parameter and postprocessing technique. For instance, the selection of pharmacokinetic model directly reflects *K_trans_* values: if the acquisition duration is short and the rate of BBB leakage estimates are slow, the Patlak model that does not allow back-flow into the capillary will be suitable, while the conventional Tofts and the extended Tofts models can result in increased uncertainty in the fitted parameters (Heye and Culling, 2014). Since contrast agents have relatively large molecular weights (Gd-DTPA 550 Da), BBB permeability necessarily reaches beyond a physiological level before extravasation occurs (Shao et al., 2020). Hence, DCE-MRI with the Patlak model analysis has been increasingly used to quantify low-level BBB permeability in patients with AD pathological changes. An overview of DCE-MRI studies for subjects with the AD continuum is summarized in Table 2 (Starr et al., 2009; Anderson et al., 2011; Montagne et al., 2015; van de Haar et al., 2016a,b, 2017; Montagne et al., 2019; Nation et al., 2019; Dickie et al., 2020; Freeze et al., 2020; Montagne et al., 2020; Chagnot et al., 2021; Li et al., 2021; Choi et al., 2022). As mentioned above, there are considerable differences in *K_trans_* values among the studies. Note that measuring BBB permeability with DCE-MRI would be confounded by several factors that should be considered when acquiring or interpreting such data. Especially in studies of AD that involve subtle BBB permeability, the modifications implemented to improve the accuracy of *K_trans_* values must be considered (Manning et al., 2021). As another matter of note, the invasiveness of the injection of gadolinium-based contrast agents and the contraindication for patients with renal insufficiency who might possibly develop nephrogenic systemic fibrosis should be noted. To err on the safe side, the DCE-MRI methodology must be guided by the risk–benefit ratio (Montagne et al., 2016).

**Table 2:** Overview of DCE-MRI studies in subjects with the AD continuum.

Study	Subjects: sample size/age/diagnosis	Image acquisition parameters	Pharmacokinetic model	*K_trans_* (× 10^−3^ min^−1^)	Main findings
Wang et al. (2011)	11/74 ± 7/MCI	Philips, 1.5 T, GRE, axial, 8 mm thickness	Signal enhancement ratio	NA	The first reported DCE-MRI study in MCI. BBB leakage is increased in MCI
Starr et al. (2009)	15/73.7/AD	GE, 1.5 T, FSPGR, axial, 3 mm thickness, 30 min	Signal enhancement ratio	NA	The first reported DCE-MRI study in AD. Temporal signal intensity pattern differed
Anderson et al. (2011)	1/71/early AD	Siemens, 7 T, TurboFLASH, axial	Two-compartment exchange model	NA	BBB water regulation is disturbed in AD and results in abnormal BBB permeability
Montagne et al. (2015)	20/55–85/MCI	GE, 3 T, FSPGR coronal, voxel size = 0.625 × 0.625 × 5 mm^3^, 16 min	Patlak model	1.49 ± 0.31 (SFG)	BBB permeability contributes to cognitive impairment in aging and MCI
				1.27 ± 0.25 (ITG)	
				2.30 ± 0.36 (WM)	
van de Haar et al. (2016a)	16/59–85/early AD	Philips, 3 T, Dual-time SRGRE, axial, voxel size = 1 × 1 × 5 mm^3^, 25 min	Patlak model	0.089 ± 0.112 (GM)	BBB permeability is associated with cognitive decline in patients with early AD
				0.066 ± 0.044 (WM)	
van de Haar et al. (2016b)	16/65–85/early AD	Philips, 3 T, Dual-time SRGRE, axial, voxel size = 1 × 1 × 5 mm^3^, 25 min	Patlak model	0.27 ± 0.14 (GM)	BBB permeability is increased in early AD patients, which is linked to reduced CBF
van de Haar et al. (2017)	16/73.6 ±7.9/early AD	Philips, 3 T, Dual-time SRGRE, axial, voxel size = 1 × 1 × 5 mm^3^, 25 min	Patlak model	0.104 ± 0.124 (GM)	BBB permeability is higher in patients with early AD
				0.075 ± 0.046 (WM)	
Montagne et al. (2019)	12/75/MCI	GE, 3 T, FSPGR	Patlak model	NA	BBB permeability is increased in MCI
		coronal, voxel size = 0.625 × 0.625 × 5 mm^3^, 16 min			
Nation et al. (2019)	20/73/MCI	GE, 3 T, FSPGR	Patlak model	1.35 (GM)	BBB permeability is increased in MCI, independent of Aβ and tau pathology
		coronal, voxel size = 0.625 × 0.625 × 5 mm^3^, 16 min		2.39 (WM)	
Freeze et al. (2020)	34/71.6 ± 6.7/AD	Philips, 3 T, Dual-time SRGRE, axial, voxel size = 1 × 1 × 2 mm^3^, 25 min	Patlak model	7.4 × 10^−4^ (GM)	BBB permeability is related to CSVD severity in AD patients
				8.1 × 10^−4^ (WM)	
Montagne et al. (2020)	39/72/MCI	Philips or Siemens, 3 T, VIBE with variable flip angle, coronal, voxel size = 0.55 × 0.55 × 5 mm^3^, 16 min	Patlak model	1.42 (GM)	BBB permeability is increased in APOE4 carriers
				2.13 (WM)	
Li et al. (2021)	26/71.04 ± 8.99/MCI	Siemens, 3 T, SPGR with variable flip angle, axial, voxel size = 1.2 × 1.2 × 3 mm^3^	Patlak model	0.157 ± 0.07 (GM)	BBB permeability is increased in patients with vascular cognitive impairment
				0.031 ± 0.014 (WM)	

Choi et al. (2022)	147/76 ± 8/AD	Siemens, 3 T, GRE, coronal, voxel size = 1.25 × 1.25 × 3 mm^3^	Patlak model	0.37 (Choroid Plexus)	BBB permeability is inversely correlated with the volume of choroid plexus

Aβ, β-amyloid; AD, Alzheimer’s disease; APOE4, apolipoprotein E ɛ4; BBB, blood–brain barrier; CSVD, cerebral small vessel disease; DCE-MRI, dynamic contrast-enhanced magnetic resonance imaging; FLASH, fast low-angle shot; FSPGR, fast spoiled gradient-echo; IFG, inferior frontal gyrus; GM, gray matter; GRE, gradient echo; MCI, mild cognitive impairment; NA, not applicable; SFG, superior frontal gyrus; SPGR, spoiled gradient; SRGRE, saturation recovery gradient echo; VIBE, volumetric interpolated breath-hold; WM, white matter.

### 5.2. Overview of arterial spin labeling-based blood–brain barrier imaging in subjects with the Alzheimer’s disease continuum

In ASL-based BBB imaging, water is an endogenous tracer alternative to contrast agents, and has a much smaller molecular weight (≈ 18 Da). Therefore, assessing the BBB water exchange rate using the ASL-based BBB imaging can provide a more sensitive assessment of BBB dysfunction at the earliest stages of AD. A recent review has comprehensively summarized the mechanisms of water exchange rate across the BBB, acquisition methods, and mathematical models (Dickie et al., 2020). ASL is a non-invasive technique with which to measure cerebral blood flow, and kinetic models have been proposed to map the transvascular water exchange rate based on the T2 (Ohene et al., 2019) or diffusion coefficient (Shao et al., 2019) differences between the intra-and extravascular compartments. Regional water exchange rate across the BBB can be quantified based on the kinetic modeling of ASL signals in the two compartments. Clinical studies have shown that an altered BBB water exchange rate is associated with aging (Li et al., 2005; Anderson et al., 2020), multiple sclerosis (Rooney et al., 2015), and obstructive sleep apnea (Palomares et al., 2015). In a cohort of early-stage AD subjects who were categorized into apolipoprotein E genotyping, an apolipoprotein E *ɛ4* dose was associated with a decreased BBB water exchange rate, resulting in brain Aβ accumulations and cognitive disturbances (Uchida et al., 2022a). An overview of ASL-based BBB imaging studies for subjects with the AD continuum is summarized in Table 3 (Shao et al., 2019; Joseph and Novel, 2020; Shao et al., 2020; Gold et al., 2021; Ford et al., 2022; Uchida et al., 2022a).

**Table 3:** Overview of ASL-based BBB imaging in subjects with the AD continuum.

Study	Subjects: Sample Size/Age/Diagnosis	Image acquisition parameters	Water exchange model	*k_w_* (min^−1^)	Main findings
Shao et al. (2019)	19/68.8 ± 7.6/Cognitively normal and MCI	Siemens, 3 T, DP-pCASL with 3D GRASE, axial, voxel size = 3.5 × 3.5 × 8 mm^3^, 6 min	Regularized SPA	98.3 ± 20.8 (Frontal lobe)	BBB water exchange rate is associated with vascular risk factors and cognitive scores
				97.8 ± 17.3 (Temporal lobe)	
				100.6 ± 22.2 (Parietal lobe)	
Shao et al. (2020)	16/62–86/Cognitively normal and MCI	Siemens, 3 T, DP-pCASL with 3D GRASE, axial, voxel size = 3.5 × 3.5 × 8 mm^3^, 5.53 min	Regularized SPA	122.3 ± 16.5 (Whole brain)	Only three brain regions have correlations between BBB water exchange *k_w_* and BBB permeability *K_trans_*
				122.6 ± 15.6 (Gray matter)	
				121.9 ± 17.2 (White matter)	
Joseph and Novel (2020)	3/65–85/mild AD	Siemens, 3 T, TGSE PASL, axial, voxel size = 3.9 × 3.9 × 4 mm^3^, 20 min	NA	NA	Reduced paravascular clearance in mild AD
Gold et al. (2021)	39/72.7/preclinical AD	Siemens, 3 T, DP-pCASL with 3D GRASE, axial, voxel size = 3.5 × 3.5 × 8 mm^3^, 5.53 min	Regularized SPA	104.4 ± 22.2 (Frontal lobe)	Low BBB water exchange rate is associated with low CSF Aβ42 concentration
				94.8 ± 26.1 (Temporal lobe)	
				83.2 ± 28.3 (Parietal lobe)	
Ford et al. (2022)	30/25–65+/Cognitively normal and MCI	GE, 3 T, DP-pCASL with QPM, axial, voxel size = 1.9 × 1.9 × 4 mm^3^, 27 min	Regularized SPA	81.5 ± 15.4 (GM)	BBB water exchange rate is inversely correlated with age
				75.2 ± 13.9 (WM)	
Uchida et al. (2022a)	66/72.2 ± 5.8/early AD	Philips, 3 T, DP-pCASL with 3D GRASE, axial, voxel size = 3.5 × 3.5 × 7 mm^3^, 14 min	Regularized SPA	95.1 ± 7.32 (Frontal lobe)	BBB water exchange rate is decreased in APOE4 carriers, resulting Aβ accumulations
				91.3 ± 7.05 (Temporal lobe)	
				94.5 ± 7.19 (Parietal lobe)	

CSF, cerebrospinal fluid; DP-pCASL, diffusion-prepared pseudo-continuous arterial spin labeling; GRASE, gradient and spin-echo; PASL, pulsed arterial spin labeling; QPM, quantitative permeability mapping; SPA, single-pass approximation; TGSE, turbo gradient spin echo.

## 6. Other Alzheimer’s disease pathophysiology in relation to blood–brain barrier imaging

### 6.1. Aging

Aging is the most common cause of AD pathogenesis, and BBB dysfunction is a hallmark of aging and aging-related disorders, including AD and CSVD (Ford et al., 2022). Converging evidence suggests that BBB dysfunction plays a central role in the aging brain (Weiss et al., 2009; Banks et al., 2021). BBB permeability *K_trans_* values were increased with normal aging (Montagne et al., 2015) and associated with age-related cognitive dysfunction (Bowman et al., 2018; Nation et al., 2019), which was the most prominent in the hippocampus (Montagne et al., 2015; Ivanidze et al., 2019). These *K_trans_* values measured by the DCE-MRI analysis primarily reflect the paracellular leakage of the relatively large gadolinium contrast agents through injured endothelial tight junctions (Laurent et al., 2006; Wang et al., 2011). Meanwhile, ASL-based BBB imaging can capture the transcellular transport of the much smaller water molecules through AQP4 channels on astrocytic end-feet. Decreased AQP4 polarization was associated with aging and Aβ brain deposition in animal models (Yang et al., 2011; Kress et al., 2014; Ishida et al., 2020) and in human brain tissues (Zeppenfeld et al., 2017). In accordance with these basic experiments, a recent paper found that BBB water exchange rate *k_w_* values, measured by DP-pCASL with Quantitative Permeability Mapping analysis *in vivo*, demonstrated a negative correlation with aging, with adjustments for gender and the number of vascular risk factors as covariates (Ford et al., 2022).

### 6.2. Vascular risk factors

A close relationship between AD pathogenesis and vascular risk factors, such as hypertension, diabetes, hyperlipidemia, smoking, and lack of exercise, is supported by cumulative evidence (Viswanathan et al., 2009). These vascular risk factors lead to BBB dysfunctions that are associated with AD and vascular cognitive impairments (Yang et al., 2022). BBB imaging is useful for estimating to what extent the BBB function would be damaged at the individual level (Thrippleton et al., 2019), and is part of the design of a therapeutic trial to control vascular risk factors for the prevention of cognitive decline (Chagnot et al., 2021). BBB permeability *K_trans_* values, using DCE-MRI in patients with diabetes, were increased in white matter, which is reflective of cerebral microangiopathy, before CSVD features, such as lacunes, microbleeds, and white matter hyperintensities, could be visualized on conventional MRI (Chen et al., 2021). BBB water exchange rate *k_w_* values using DP-pCASL in patients with vascular risk factors were positively correlated with white matter hyperintensity severity and negatively with executive/episodic memory scores (Shao et al., 2019).

### 6.3. White matter hyperintensity

White matter hyperintensities are common findings in the elderly population and generally considered ischemic lesions related to CSVD (Yamanaka et al., 2019). CSVD covers a wide array of pathologies involving the dysfunction of the cerebral small vessels. Clinical manifestations include recurrent stroke, cognitive impairment, and gait disturbance. CSVD is a common cause of dementia, with characteristic broadened white matter hyperintensities. While the pathogenesis of white matter hyperintensity remains unclear, BBB leakage is one of the most accepted hypotheses due to its strong association with white matter hyperintensity (Wardlaw et al., 2016; Li et al., 2017; Kerkhofs et al., 2021). Notably, normal-appearing white matter tissues surrounding white matter hyperintensities presented increased BBB permeability, suggesting that an abnormality on BBB imaging could precede further extensions of these white matter lesions (Shao et al., 2020). Because DCE-MRI-based differences in BBB leakage due to white matter lesions is very subtle (Zhang et al., 2017), the Patlak model is recommended to elucidate these differences (Thrippleton et al., 2019). Several reviews have summarized the mechanism of BBB disruption, offered recommendations for BBB imaging analyses, and interpretations of BBB abnormalities, particularly in the white matter, in patients with CSVD (Heye and Culling, 2014; Thrippleton et al., 2019; Dickie et al., 2020; Chagnot et al., 2021).

### 6.4. Cerebrospinal fluid

Cerebrospinal fluid, predominantly produced in the ventricles and circulating throughout the brain, fills the perivascular space and interacts closely with BBB function (Nakada and Kwee, 2019). The production, circulation, and clearance of cerebrospinal fluid have crucial pathophysiological implications for brain diseases. It also plays a role in the clearance of Aβ and tau proteins to protect from AD pathogenesis (Selkoe and Hardy, 2016). To support this protective function, reduced cerebrospinal fluid production and clearance caused exacerbated AD pathologies (Silverberg et al., 2001; Tarasoff-Conway et al., 2015). Several neuroimaging modalities have been developed to measure cerebrospinal fluid dynamics (Mehta et al., 2022). MR cisternography and MR myelography were conventionally used for the diagnosis of anatomical cerebrospinal fluid disorders (Mokri, 2014). Extending beyond the leakage, phase-contrast cine MRI is the most widely used imaging modality for cerebrospinal fluid dynamics, which is a non-invasive technique without the need for contrast administration or catheterization (Barkhof et al., 1994). Decreased cerebrospinal fluid flow using phase-contrast cine MRI analysis was associated with cognitive deficits in elderly individuals (Attier-Zmudka et al., 2019).

### 6.5. Oxygen extraction fraction

In addition to the interstitial and cerebrospinal fluid dynamics, pre-and post-capillary vessels in the brain are also key factors in AD pathogenesis. Close monitoring of arterial and venous blood oxygenation serves as a novel biomarker for the study of cerebral hemodynamics (Lu and Ge, 2008), which can aid in understanding the NVU pathophysiology. Oxygen extraction fraction is a physiologic marker that reflects the percentage of oxygen extracted from the blood supply of the brain, which is directly associated with brain oxygen metabolism (Buxton et al., 2004). The oxygen extraction fraction shows a pronounced increase with aging in cognitively healthy individuals (Peng et al., 2014). Meanwhile, oxygen extraction fraction in cognitively impaired individuals has shown various results due to the etiology-based diagnosis of cognitive impairment. For instance, AD pathophysiology led to diminished neural activities, and thereby, decreased oxygen extraction fraction in patients with AD (Butterfield and Halliwell, 2019). Conversely, CSVD pathophysiology caused a reduction in blood supply and resulted in an elevated oxygen extraction fraction (Jann et al., 2021). Thus, these findings were useful in the differential diagnosis for AD and vascular cognitive impairment (Jiang et al., 2020). Longitudinal changes in an elevated oxygen extraction fraction in older adults were associated with the progression of vascular risk factors and white matter hyperintensity volumes, independent of the AD pathologies (Lin et al., 2022).

### 6.6. Brain iron

The homeostasis and physiological role of brain iron in AD and CSVD has been debated for decades (Tao et al., 2014). QSM has been used to detect the abnormal iron deposition in each specific region as a clinical application and quantify the iron concentration for clinicoradiological research (Acosta-Cabronero et al., 2013; Ayton et al., 2017; Kim et al., 2017; Tiepolt et al., 2018; Gong et al., 2019; Uchida et al., 2020, 2022a,b; Cogswell et al., 2021). Although abnormally high levels of iron are thought to induce free radicals, resulting in neuronal loss and cognitive dysfunction, whether iron deposition is a cause or a result of Alzheimer’s pathogenesis remains elusive. The former hypothesis that brain iron would play a role in the cause of AD is supported by combined QSM and BBB imaging studies, which revealed iron leakage owing to BBB disruption in CSVD using DCE-MRI (Mikati et al., 2014; Tariq et al., 2018; Uchida et al., 2020), and subtle BBB dysfunction in early stages of the AD continuum with the *ɛ4* allele of the *APOE* gene using DP-pCASL (Uchida et al., 2022a). Meanwhile, cerebral microbleeds are frequently observed as an incidental finding, or in the context of an associated Alzheimer’s pathologic finding, such as cerebral amyloid angiopathy (Haller et al., 2018). Consequently, brain iron perturbations detected by QSM could be valuable monitoring tools during AD pathological processes (Fazlollahi et al., 2020; Rotta et al., 2021).

## 7. Challenges, expectations, and future directions

### 7.1. Challenges

Challenges and pitfalls exist in the measurement of BBB function to capture AD pathophysiology using magnetic resonance-based BBB imaging technologies. In the DCE-MRI analysis, paracellular BBB leakage of low-molecular-weight gadolinium contrast agents is tracked dynamically as these agents pass from the intravascular to the extravascular space (Heye and Culling, 2014). This approach can detect only major damage to endothelial tight junctions such that gadolinium leaks out of the BBB. Therefore, it cannot detect abnormalities in early AD where the BBB damage has not yet reached the threshold of gadolinium leakage through the endothelium. In addition, it is difficult to find specific transporter alterations, such as AQP4, because the contrast agents are not specifically designed to trace BBB functions (Dickie et al., 2020). In ASL-based BBB imaging, it is difficult to measure anatomical details because of its low spatial resolution and signal-to-noise ratio when acquiring the ASL images (Dickie et al., 2020). Low spatial resolution also causes partial volume errors, whereas the AIF definition on the superior sagittal sinus with a coronal section in the DCE-MRI analysis minimizes these errors (Chagnot et al., 2021). Further technical limitations of these BBB imaging analyses should be highlighted: Gibbs ringing; signal drift; patient motion; AIF definition errors; and kinetic model inaccuracy can confound measurements due to the low amplitude of signal changes (Thrippleton et al., 2019; Chagnot et al., 2021).

### 7.2. Expectations

While it has long been recognized that water does not diffuse freely across the BBB (Eichling et al., 1974; Bolwig and Lassen, 1975; Herscovitch et al., 1987; Takagi et al., 1987), the idea that water could be used as an internal tracer for measuring BBB function has only recently been proposed (Dickie et al., 2020). In addition to potential safety benefits with no concerns for accumulation of gadolinium in the brain, the use of water to probe BBB function in AD pathophysiology has the following expectations: 1) due to the small size of a water molecule, subtle BBB alterations are likely to be detectable at an earlier stage during the AD pathogenesis; 2) water is physiologically transported across the BBB by both passive and active pathways through co-transporters and uniporters (Zeuthen, 2010) between the capillary, cerebrospinal, and interstitial fluids, potentially providing a wide range of BBB pathophysiology; 3) water has its own transport protein (AQP4) within the NVU system, which closely correlates with the glymphatic flow involved in clearing interstitial solutes (Iliff et al., 2012).

### 7.3. Future directions

A number of MRI techniques with which to measure the BBB water exchange rate have been developed in addition to DP-pCASL. Recently, a motion-compensated diffusion-weighted pCASL was proposed to acquire intravascular/extravascular perfusion signals from multiple PLDs. Using three-compartment SPA modeling, signal-to-noise ratio increased three-fold and spatial resolution achieved 3.5mm^3^ isotropic (Shao et al., 2023). Additionally, a multi-echo ASL method was developed to improve the ability to distinguish intravascular-and extravascular-labeled water (Wells et al., 2009). The principal of this method to quantify the BBB water exchange rate depends entirely on intrinsic R2 (= 1/T2) differences between the multi-compartmental origin of labeled water (Wells et al., 2013). If a reliable estimation of the BBB water exchange rate using the multi-echo ASL analysis can be achieved, spatial resolution and the signal-to-noise ratio of the *k_w_* map will be improved (Gregori et al., 2013). Furthermore, the water extraction-with-phase-contrast-arterial-spin-tagging (WEPCAST; Lin et al., 2018) and magnetization transfer-weighted ASL methods (Silva et al., 1997) take unique approaches to the measurement of the BBB water exchange rate. The former measures *PS_w_* by quantifying the transmitted fraction of labeled water that passes into the superior sagittal sinus during a single pass (Lin et al., 2018). The latter can separate intravascular and extravascular signals based on their different magnetization transfer effects because macromolecular spins interact more strongly with extravascular water (Silva et al., 1997). Since there has been, as yet, no gold standard model with which to measure the BBB water exchange rate, a direct comparison of the *k_w_* values among these methodologies will be needed.

In addition to the cross-sectional design of much of the BBB imaging research, longitudinal clinical and radiological studies for individuals who are suspected to be on the AD continuum should be performed. Such studies could reveal the onset of BBB dysfunction, the relationship between BBB imaging alterations and the Alzheimer’s pathology and cognitive decline, and the potential opportunity for timely therapeutic and preventive interventions. ASL-based BBB imaging, which makes use of water as an internal tracer to estimate the BBB function, fits well with these longitudinal clinical and radiological studies so that individuals with the AD continuum can undergo repeated follow-up examinations without concern about adverse effects.

## 8. Conclusion

Magnetic resonance-based BBB imaging, such as DCE-MRI and DP-pCASL, has already contributed significantly to a better understanding of AD pathogenesis in relation to NVU pathophysiology. Despite being more broadly used, we also highlighted the technical limitations of each BBB imaging model. Nevertheless, we conclude that these magnetic resonance-based BBB imaging methodologies are unique and useful applications that reflect the pathophysiological properties between the interstitial, cerebrospinal, and capillary fluids in the central nervous system and have the potential to measure the efficacy of future BBB-targeted therapeutics in clinical settings for AD and related dementias.

## Author contributions

YU: conceptualization, investigation, data curation, writing—original draft, and funding acquisition. HK: conceptualization, data curation, and writing—review and editing. KS: data curation and writing—review and editing. KO: supervision and writing— review and editing. NM: conceptualization, supervision, and writing— review and editing. All authors contributed to the article and approved the submitted version.

## Funding

This work was supported by Grants-in-aid of the 24th General Assembly of the Japanese Association of Medical Sciences, Reiwa 3 Grants-in-aid for Young Scientists of the Kowa Life Science Foundation, Grants-in-aid from the 2021 Japan Brain Foundation, and KAKENHI Grant-In-Aid for Scientific Research C (22 K07520).

## Conflict of interest

KO is a consultant for “AnatomyWorks” and “Corporate-M.” This arrangement is being managed by the Johns Hopkins University in accordance with its conflict-of-interest policies.

The remaining authors declare that the research was conducted in the absence of any commercial or financial relationships that could be construed as a potential conflict of interest.

## Publisher’s note

All claims expressed in this article are solely those of the authors and do not necessarily represent those of their affiliated organizations, or those of the publisher, the editors and the reviewers. Any product that may be evaluated in this article, or claim that may be made by its manufacturer, is not guaranteed or endorsed by the publisher.
